# Striatum-Centered Fiber Connectivity Is Associated with the Personality Trait of Cooperativeness

**DOI:** 10.1371/journal.pone.0162160

**Published:** 2016-10-18

**Authors:** Xuemei Lei, Chuansheng Chen, Chunhui Chen, Qinghua He, Robert K. Moyzis, Gui Xue, Qi Dong

**Affiliations:** 1 School of Psychology, Beijing Normal University, Beijing, China; 2 Department of Psychology and Social Behavior, University of California, Irvine, California, United States of America; 3 National Key Laboratory of Cognitive Neuroscience and Learning, Beijing Normal University, Beijing, China; 4 School of Psychology, Southwest University, Beibei, Chongqing, China; 5 Department of Biological Chemistry, School of Medicine, University of California, Irvine, California, United States of America; University of Texas at Austin, UNITED STATES

## Abstract

*Cooperativeness* is an essential behavioral trait evolved to facilitate group living. Social and cognitive mechanisms involved in cooperation (e.g., motivation, reward encoding, action evaluation, and executive functions) are sub-served by the striatal-projected circuits, whose physical existence has been confirmed by animal studies, human postmortem studies, and *in vivo* human brain studies. The current study investigated the associations between *Cooperativeness* and fiber connectivities from the striatum to nine subcortical and cortical regions, including the amygdala, hippocampus, medial orbitofrontal cortex, lateral orbitofrontal cortex, ventrolateral prefrontal cortex, dorsolateral prefrontal cortex, posterior cingulate cortex/retrosplenial cortex, dorsal cingulate cortex, and rostral cingulate cortex. Results showed that *Cooperativeness* was negatively correlated with fiber connectivity for the cognitive control system (from the dorsal caudate to the rostral cingulate cortex and ventrolateral prefrontal cortex), but not with fiber connectivity for the social cognitive system (e.g., connectivity with the medial prefrontal cortex and amygdala). These results partially supported Declerck et al.’s (2013) cognitive neural model of the role of cognitive control and social cognition in cooperation.

## Introduction

Human being is a highly cooperative species. Researchers have investigated both cognitive and neural mechanisms involved in cooperative behaviors. Relevant cognitive processes include motivation, reward encoding, action evaluation, and executive functions in the context of social interactions [1–3]. The neural bases of these cognitive functions have been localized to the striatum and striatum-connected circuits [4–6]. For example, the amygdala and orbitofrontal cortex have been found to be responsible for the processing of economic and social reward, emotion, and motivation [7–15], the anterior cingulate cortex for processing others’ versus one’s own rewards [16], the dorsal anterior cingulate cortex for processing the intentions and/or mind state of other individuals in social cooperative behaviors [17, 18], and the lateral and dorsal prefrontal cortex for executive functions such as cognitive control and inhibitory control [19].

Recently, Declerck and colleges [20] integrated previous research and proposed a model of the neural basis of pro-social decision making. According to [20], two cognitive neural systems are involved in cooperative behaviors: the cognitive control system that processes extrinsic cooperative incentives and is sub-served by the lateral prefrontal cortex and anterior cingulate cortex, and the social cognition system that processes trust and/or threat signals and is sub-served by the medial prefrontal cortex and amygdala [20]. The cognitive control system has modulatory effects on reward processing via the signaling of extrinsic (dis)incentives (e.g. long-term benefits, building reputation, sanction and social norm, fear of punishment), and the social cognition system has modulatory effects on reward processing via the signaling of trust or threat (e.g. friend or foe, collaborator or competitor, compassion and empathy or aggression) [20].

Although much is known about the neural basis of cooperative behaviors as reviewed above, the previous studies focused on cooperative behaviors in experimental settings (as measured with Prisoner’s Dilemma Game or the Public Goods task) [11, 12, 21], much less is known about the neuroanatomical basis of human cooperativeness as a personality trait. Human *Cooperativeness* was constructed as a pro-social and altruistic character in Cloninger’s bio-psychosocial model of personality [22, 23]. It has been linked to one’s adaptation to external and complex social contexts. *Cooperativeness* can be measured with the subscale of the same name using the well-established Temperament and Character Inventory-Revised [22, 23]. People with high scores in *Cooperativeness* are other-regarding, and have been found to be empathetic, tolerant, compassionate, supportive, fair, and principled. People with low scores in *Cooperativeness* are self-regarding, self-absorbed, intolerant, critical, unhelpful, revengeful, and opportunistic.

These individual differences in *Cooperativeness* may reflect differential reliance on the two systems proposed by Declerck et al. [20]. Self-regarding individuals are more sensitive to extrinsic cooperative incentives and thus rely relatively more on cognitive control to make decisions about cooperation, whereas other-regarding individuals are more sensitive to trust signals to avoid betrayal and thus rely relatively more on the social cognition system [20]. Their differential reliance on the two systems may be linked to individual differences in fiber connectivities between the striatum (responsible for the reward processing system) and the two neural systems, either due to the neuroplasticity of long-term use of the systems or due to pre-existing individual differences in neuroanatomy.

In the current study, we examined the associations between *Cooperativeness* and striatum-projected fiber connectivity. Based on previous research as mentioned above, the striatum was chosen as the seed region and other nine subcortical and cortical regions were chosen as target regions, including the medial orbitofrontal cortex (mOFC), lateral orbitofrontal cortex (lOFC), ventrolateral prefrontal cortex (vlPFC), dorsolateral prefrontal cortex (dlPFC), posterior cingulate cortex/retrosplenial cortex (PCC), rostral cingulate cortex (rostral CC), dorsal cingulate cortex (dorsal CC), hippocampus, and amygdala. These striatum-projected fiber connections have been confirmed by animal studies, human post-mortem studies, and *in vivo* human brain studies [24, 25]. The striatum and nine target masks (see S1 Fig for anatomical locations of these masks) for each hemisphere were created based on the automated anatomical labeling template [26] and previous studies [27, 28]. Based on the neural mechanisms of cooperative behaviors [20], we hypothesized that *Cooperativeness* would be negatively correlated with anatomical connections from the striatum to the cognitive control regions, such as the rostral CC, vlPFC and dlPFC (Hypothesis 1), and positively correlated with anatomical connections from the striatum to the social cognition system such as medial prefrontal and orbitofrontal regions (Hypothesis 2). In addition, we included connectivities from the striatum to the hippocampus and PCC because the involvement of long-term memory and self-processing in social interactions [29, 30]. No specific hypotheses were proposed for them.

## Materials and Methods

### Participants

Fifty male and 79 female college students (mean age = 20.10 yrs, ranging from 19 to 22 yrs) were recruited from Beijing Normal University. All participants were Han Chinese with normal or corrected-to-normal vision and no neurological or psychiatric history. They also passed the physical and clinical examinations for all freshmen administered by the University. All participants were asked to complete the Temperament and Character Inventory-Revised (TCI-R) [22, 23]. Participants were all right-handed based on the Edinburgh Handedness Inventory [31]. Age and handedness did not differ between males and females. Participants were scanned for diffusion tensor and high-resolution 3D anatomical images. They all gave informed written consents and the study was approved by the Beijing Normal University’s Institutional Review Board.

### Image acquisition

Participants were scanned on a Siemens Trio 3T scanner with an eight-channel head coil in the Beijing Normal University Imaging Center for Brain Research. The diffusion-weighted data were acquired using a twice-refocused spin-echo EPI sequence with the following parameters: TR/TE = 7200ms/104ms, 49 transverse slices, field-of-view = 230*230mm, matrix = 128*128, slice thickness = 2.5mm, 1 direction with b-value = 0s/mm^2^, 64 directions with *b*-value = 1000s/mm^2^. The final voxel size was 1.8mm×1.8mm×2.5mm. In addition, a high-resolution 3D anatomical image was obtained using T1-weighted MP-RAGE sequence with the following parameters: TR/TE/FA = 2530ms/3.75ms/7°, FOV = 220*220mm, matrix = 256*256, slice thickness = 1mm, 128 sagittal slices). Scanning lasted 18 minutes for each participant.

### Image preprocessing

Diffusion tensor images (DTI) were processed using the FMRIB’s Diffusion Toolbox (FDT 2.0) [32] from the FMRIB’s Software Library (FSL, version 5.0.5; www.fmrib.ox.ac.uk/fsl) [33–35]. The standard pre-processing procedure was used for the probabilistic tractography of DTI data [36, 37]. Correction of the diffusion data for eddy currents and head motion was performed through the affine alignment to the no-diffusion-weighted reference volume (*b*-value = 0) [38]. Then, fitting of diffusion tensor was performed by using the DTIfit program implemented in FMRIB’s diffusion toolbox. Diffusion-weighted images were spatially normalized into the Montreal Neurological Institute (MNI) standard space with FMRIB's Linear Image Registration Tool (FLIRT) [39, 40] and FMRIB's Nonlinear Image Registration Tool (FNIRT) by individuals’ high-resolution T1-weighted structural image. Registration was confirmed by visual inspection. Then, transformation matrix and warp field from individual participants’ diffusion space to the MNI standard space, as well as inversed transformation matrix from the MNI standard space to individuals’ diffusion space, were acquired by the “convert_xfm” and “invwarp” commands.

### Seed brain region and target brain regions

We created one seed mask and nine target masks for each hemisphere from the automated anatomical labeling template [26] and previous studies [27, 28]. These masks included the following regions (the numbers in parentheses refer to AAL map values and correspond to the left hemisphere; subtract 1 for the right hemisphere; also note that some masks were combined): the striatum (72, 74), medial orbitofrontal cortex (mOFC;28, 6, 26), lateral orbitofrontal cortex (lOFC;10, 16), ventrolateral prefrontal cortex (vlPFC;14), dorsolateral prefrontal cortex (dlPFC; 14), posterior cingulate cortex/retrosplenial cortex (PCC; 68), rostral cingulate cortex (rostral CC, 32), dorsal cingulate cortex (dorsal CC,34), hippocampus (38), and amygdala (42). S1 Fig (see S1 Dataset for the brain masks) shows the anatomical locations of these brain masks. Due to the fact that probabilistic tractography must be conducted in individual participants’ diffusion space using the FMRIB Diffusion Toolbox, all masks in the MNI standard space were transformed to individuals’ diffusion space by using transformation matrix and warp field produced in the previous step and voxels within masks were assigned the value of 1 and outside voxels were assigned the value of 0. Finally, volumes of these ten masks in individuals’ diffusion space were obtained. The total intracranial volume (ICV) was obtained from high-resolution T1-weighted anatomical image by using the Freesurfer segmentation software package (http://surfer.nmr.mgh.harvard.edu/) [41].

### Tractography and seed-based classification

Tractography and seed-based classification methods were used in the current study following the well-validated protocol used in previous diffusion tensor imaging studies [27, 42–44]. For each participant, Bayesian estimation of diffusion parameters was conducted with a dual-fiber model allowing for crossing fibers by using the BedpostX program implemented in FMRIB’s diffusion toolbox [43]. Probabilistic tractography was performed from the striatum to nine target regions (mOFC, lOFC, dlPFC, vlPFC, rostral CC, dorsal CC, hippocampus, and amygdala) in individuals’ diffusion space by using PROBTRACKS program implemented in FMRIB’s diffusion toolbox [43]. Five thousand tract-following samples were initiated in each voxel of the striatum, and tracked to the nine target regions, which resulted in nine probabilistic maps of fiber connectivity (tractographic images) for the nine target regions. The value of each voxel in the tractographic image represented the number of the tracking to the target region (connectivity between the voxel in the striatum to all voxels in the target region). These nine images were thresholded by at least 10 tracking in each voxel. All images passed the threshold. The value of each voxel was then converted to proportional ratio by dividing it by the voxel’s total tracking number to all regions. The resulting nine images were transformed back to the MNI standard space for group statistical analyses. The final spatial resolution was 1 mm^3^. All preprocessing was done separately for each hemisphere. In the final step, tractographic images in the MNI standard space from the two hemispheres were combined and later used to examine correlations between *Cooperativeness* and striatum-projected fiber connectivity in general linear models.

The group-averaged tractographic image for each target region was obtained by averaging individual tractographic images across subjects, yielding nine group-averaged tractographic images. The seed-based classification image of the striatum was created by coloring each voxel in the striatum based on the group-averaged fiber connectivity.

### Statistical Analysis

Independent sample t test in SPSS was used to examine gender differences in age, handedness, personality traits, and ICV. Gender differences in volumes of the ten brain regions were analyzed by linear regression model with gender and ICV as predictors. Correlation analysis among seven temperament and character factors was also performed. We also examined whether gender interacted with the volumes of brain regions on *Cooperativeness*. We included de-meaned gender, volumes of seed and target regions (striatum, rostral CC, dorsal CC, dlPFC, vlPFC, lOFC, mOFC, PCC, hippocampus, and amygdala), total intracranial volume (ICV), and interaction items of gender×brain volumes as predictors.

To find sub-regions of the striatum where fiber connectivities with target regions showed significant correlations with *Cooperativeness*, we conducted voxel-wise analysis with the general linear model by using the “randomize” program in FSL [45]. In the general linear model (GLM), the *Cooperativeness* score was the covariate of interest, and gender and ICV were the confounding variables. The permutation-based non-parametric method with the Threshold-Free Cluster Enhancement (TFCE) [46, 47] was used to find significant clusters. We further extracted the average value of fiber connectivity within significant regions for each participant to visualize the correlations between *Cooperativeness* and fiber connectivity within the specific regions, and examined the interaction of gender×fiber connectivity on *Cooperativeness*.

## Results

Correlations among the seven factors/dimensions in TCI-R were consistent with previous studies [48, 49]. *Cooperativeness* was positively correlated with *Reward Dependence* (*r* = 0.455, *p* < 0.01) and *Self-directedness* (*r* = 0.413, *p* < 0.01), and negatively correlated with *Harm Avoidance* (*r* = ‒0.311, *p* < 0.01).

Gender differences in temperaments, characters, volumes of the ten brain regions (e.g. mOFC, lOFC, dlPFC, vlPFC, rostral CC, dorsal CC, PCC, hippocampus, and amygdala; see S1 Fig for anatomical locations of these regions), and ICV are shown in Table 1 (see S1 Table for dataset). There were no significant gender differences in age, handedness, temperaments, and characters (*p*s > 0.05). Males had larger ICV compared to females. With and without controlling the ICV, males consistently showed significantly larger brain regions than did females. Regression model with de-meaned *Cooperativeness*, gender, volumes of the ten brain regions, ICV, and gender × brain interaction did not reach significance (*F*_(23, 128)_ = 1.197, *p* = 0.264), which suggested no significant main effects or interactions between gender and volumes of the brain regions.

**Table 1:** Descriptive statistics and gender differences in demographical, behavioral, and brain measures.

	All (N = 129)		Male (N = 50)		Female (N = 79)		Statistics	
Variables	Mean	SD	Mean	SD	Mean	Mean	*T*	*p*
**Demographic variables**								
Age	19.93	0.95	20.10	1.00	19.80	0.94	1.67	0.15
Handedness^a^	90.99	11.02	90.28	11.34	91.44	10.04	-0.61	0.54
**Temperaments and Characters**								
*Cooperativeness*	125.49	12.85	124.22	13.88	126.29	12.17	-0.89	0.37
*Novelty seeking*	101.53	11.41	99.20	12.56	103.00	10.43	-1.86	0.07
*Reward dependence*	99.08	10.99	96.72	9.96	100.57	11.40	-1.96	0.06
*Persistence*	114.11	14.77	116.68	15.83	112.48	13.92	1.58	0.12
*Harm avoidance*	97.71	15.51	98.18	16.05	97.42	15.25	0.27	0.79
*Self-directedness*	124.34	16.34	123.72	15.95	124.73	16.66	-0.34	0.73
*Self-transcendence*	75.70	11.94	77.28	12.04	74.70	11.84	1.12	0.23
**Volumes of Brain**^b^								
dlPFC	101601.8	11576.5	107762.7	11174.7	97702.5	10086.9	-4.71	<.0001
vlPFC	28315.1	4851.6	30336.4	4809.9	27035.8	4451.5	-3.41	0.001
lOFC	32133.7	3217.6	33755.7	3277.2	31107.2	2736.5	-4.51	<.0001
mOFC	30932.9	3566.8	33020.1	3100.7	29611.9	3205.8	-5.27	<.0001
Rostral CC	16046.1	2607.6	17262.9	2613.4	15276.0	2306.0	-3.74	<.0001
Dorsal CC	25764.2	2483.0	27156.9	2375.5	24882.7	2131.2	-5.03	<.0001
PCC	42031.1	4901.4	44869.1	4624.3	40234.9	4190.3	-5.08	<.0001
Amygdala	2841.3	371.3	3042.7	369.2	2713.9	313.3	-5.09	<.0001
Hippocampus	11770.0	896.6	12343.5	929.0	11407.0	657.8	-6.03	<.0001
Striatum	23843.3	2325.4	25192.8	1930.8	22989.2	2150.8	-5.04	<.0001
ICV	1459216.8	231556.24	1543662.4	207768.4	1405770.2	231098.4	-3.43	0.001

Abbreviations: ICV, intracranial volume; dlPFC, the dorsolateral prefrontal cortex; vlPFC, the ventrolateral prefrontal cortex; mOFC, the medial orbitofrontal cortex; lOFC, the lateral orbitofrontal cortex; rostral CC, the rostral cingulate cortex; dorsal CC, the dorsal cingulate cortex; PCC, the posterior cingulate cortex/retrosplenial cortex.

^a^Determined using Edinburgh Inventory [31]; Scores greater than 0 indicate right-handedness. A score of 100 indicates strong right-handedness.

^b^ For specific regions, ICV was controlled for.

**Fig 1:**
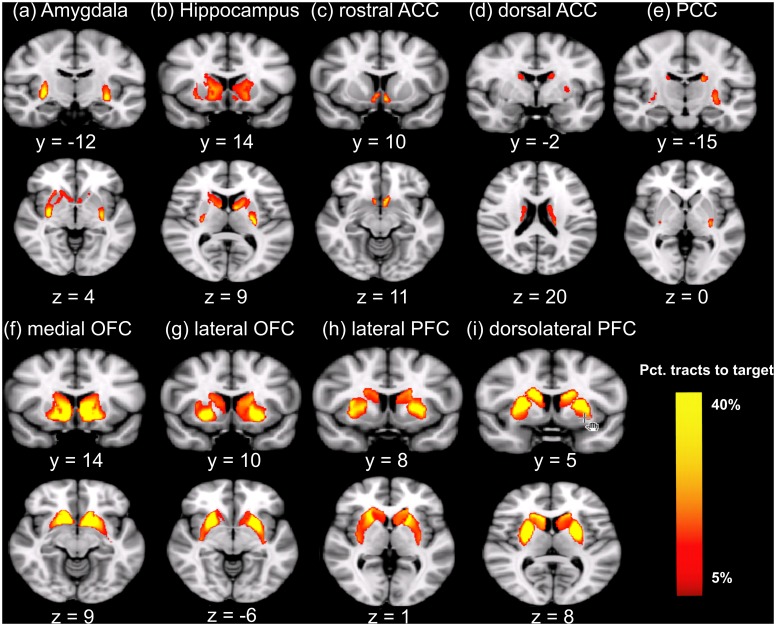
Tracts into the striatum from each target region. The color value at each voxel indicates the proportion of tracts that begin at that voxel and end in the specified target region, compared to the total number of tracts that begin at the voxel and end in any of the target regions. Only voxels with at least 5% target-ending tracts are displayed. Fig 1 is modified from one of author's published articles, which used the same tracking analysis [26]. Abbreviations: mOFC, the medial orbitofrontal cortex; lOFC, the lateral orbitofrontal cortex; vlPFC, the ventrolateral prefrontal cortex; dlPFC, the dorsolateral prefrontal cortex; PCC, the posterior cingulate cortex/retrosplenial cortex; dorsal CC, the dorsal cingulate cortex; rostral CC, the rostral cingulate cortex; Amy, amygdala; Hipp, hippocampus; CO, *Cooperativeness*.

Tracts into the striatum from each target regions are shown in Fig 1 (see S2 Dataset for nine group-average tractographic images). Apparently, different sub-regions of the striatum were differentially connected to the nine target regions. Clear anterior-posterior, medial-lateral, and dorsal-ventral connectivity patterns were observed, with the ventral and medial striatum showing stronger connections with rostral CC, mOFC, and lOFC, whereas the dorsal and lateral striatum showing stronger connections with dorsal CC, dlPFC, and vlPFC. The most posterior putamen was connected with the amygdala and hippocampus, whereas the posterior caudate was connected with PCC. These results were consistent with the segmentation pattern reported in previous studies [27, 50–52], which confirmed the validity of probabilistic tracking of diffusion tensor images in the current study.

**Fig 2:**
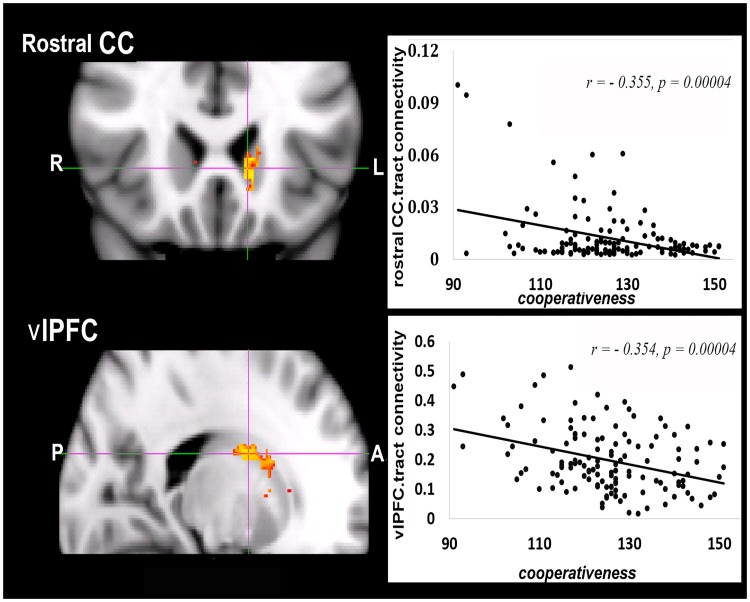
Fiber tracking from the caudate to the rostral CC and vlPFC predicted *Cooperativeness*. The coordinates in Fig 2 are the peaks within the significant regions. Tract strengths on the right side of Fig 2 are the average values extracted from the significant sub-regions of caudate shown on the left side of Fig 2. Abbreviations: rostral CC, the rostral cingulate cortex; vlPFC, ventrolateral prefrontal cortex; CO, *Cooperativeness*.

General linear analysis in the “randomize” models for the whole sample revealed significant negative correlations between *Cooperativeness* and fiber connectivity from the caudate projected to rostral CC and vlPFC (Fig 2 and Table 2). There were no significant associations between *Cooperativeness* and fiber connectivities from the striatum to the amygdala, hippocampus, PCC, dACC, dlPFC, mOFC, and lOFC (see S3 Dataset for statistic maps of nine target regions). As shown on the right side of Fig 2, *Cooperativeness* was negatively correlated with caudate-rostral CC fiber connectivity (*r* = ‒0.355, *p* = 0.0004) and caudate-vlPFC fiber connectivity (*r* = ‒0.354, *p* = 0.00004). The sub-region of the caudate that was connected to the rostral CC was located to the interface of the head of the caudate and posterior caudate, whereas the sub-region of the caudate connected to the vlPFC was located in the posterior caudate. Individuals with weaker fiber connectivities from the caudate to rostral CC and vlPFC had higher scores on *Cooperativeness*.

**Table 2:** Significant associations between *Cooperativeness* and fiber connectivity.

Fiber-originating Regions	Fiber-terminating Regions	Cluster Volume/mm^3^	Tmax	MNI Coordinates	*r*	*p*^a^
All individuals (N = 129)						
Rostral CC^b^	Left caudate	1923	3.92	[-15, 24, 2]	- 0.355	.0004
vlPFC^c^	Left caudate	318	3.97	[-19, 1, 24]	- 0.354,	.00004

^a^Significance level was adjusted for multiple tests of nine target regions (*p* = 0.05/9 = 0.004).

^b^rostral CC, the rostral cingulate cortex

^c^vlPFC, the ventrolateral prefrontal cortex

Fiber connectivities were extracted from the caudate where fiber connectivity to the rostral CC and vlPFC showed significant correlations with *Cooperativeness*. The regression model with demeaned gender, extracted caudate-rostral CC and caudate-vlPFC fiber connectivities, and gender × fiber connectivity interactions items as predictors was used to examine gender effects. The regression model was significant (*F*_(5, 128)_ = 6.492, *p* < 0.0001, *R*^2^ = 0.209). As expected, the main effects of extracted caudate-rostral CC fiber connectivity (*Beta* = ‒0.303, *t* = ‒2.74, *p* = 0.007) and caudate-vlPFC fiber connectivity (*Beta* = ‒0.298, *t* = ‒3.47, *p* = 0.001) on *Cooperativeness* were significant. There were no other significant main effects of gender (*p* = 0.658), and no significant gender × connectivity interaction (for rostral CC, *p* = 0.93; for the vlPFC, *p* = 0.98). These results suggested no confounding effects from gender on the correlations between *Cooperativeness* and both caudate-rostral CC fiber connectivity and caudate-vlPFC fiber connectivity. Together, fiber connectivities from the caudate to the rostral CC and vlPFC accounted for 20.9% of variance of *Cooperativeness*.

## Discussion

The current study used the noninvasive diffusion tensor imaging technique to investigate the associations between *Cooperativeness* and fiber connectivity from the striatum to nine target regions including the PCC, dorsal CC, rostral CC, mOFC, lOFC, dlPFC, vlPFC, hippocampus, and amygdala. Based on Declerck et al.’s model [20], we proposed two hypotheses: *Cooperativeness* would be negatively correlated with anatomical connections from the striatum to the cognitive control regions including dorsal CC, rostral CC, vlPFC, and dlPFC (Hypothesis 1), and positively correlated with anatomical connections from the striatum to the social cognition system including the amygdala, mOFC, and lOFC (Hypothesis 2).

Consistent with our Hypothesis 1, results showed that individuals with stronger fiber connectivity from the caudate to rostral CC and vlPFC had lower scores in *Cooperativeness*. Fiber connectivities from the caudate to the vlPFC and rostral CC together accounted for 20.9% of the variance of *Cooperativeness*. Strong and efficient functional coupling between the caudate and lateral prefrontal cortex has been associated with better cognitive control [53], which, according to Declerck et al. [20], is needed for self-regarding individuals (i.e., low altruistic cooperativeness) to cooperate. Consistently, studies found that most individuals need to make an effort to overcome selfish impulse in social decision making [54], whereas self-regarding individuals (low *Cooperativeness)* need a greater effort (i.e., increased activity in the cognitive control system) to achieve the same level of behavioral performance [55]. Our results also implied that anatomical connections from the caudate to rostral CC and vPFC might be positively associated with the selfishness trait, which is opposite to the altruistic *Cooperativeness*. It should be noted that no significant associations were observed for the dorsal CC and dlPFC. One explanation is that these areas have other specialized functions beyond monitoring social interactions. Specifically, compared to the rostral CC, the dorsal CC has been linked to other functions such as motor control, pain perception, and error detection [56]. The dlPFC has been found to represent relationships between more complex/abstract rules for action and desired outcomes [57–59].

Contrary to our Hypothesis 2, results showed no significant associations between *Cooperativeness* and connections from the striatum to the social cognition regions, including the amygdala, mOFC, and lOFC. The orbitofrontal cortex has been implicated in nearly every function known to cognitive neuroscience and in most neuropsychiatric diseases [9]. Its functional heterozygosity might blunt the potential associations between *Cooperativeness* and connectivity from the striatum and orbitofrontal cortex. The amygdala, on the other hand, is specialized in all aspects of emotion processing [7]. Its relations to interpersonal cooperation need further research.

In addition to testing the two model-based hypotheses, we investigated the association between *Cooperativeness* and connections from the striatum to PCC and hippocampus, and found no significant results. The hippocampus and PCC are responsible for general memory and self- processing, which are necessarily involved in social interactions [29, 30], but these regions may play a less central role in cooperation as compared to other brain regions.

The current study had several limitations that should be noted. First, *Cooperativeness* as measured in the current study refers to a character that reflects one’s inclination to cooperate kindly and altruistically with others. It has all the limitations associated with a self-report measure. Future research should combine both trait and behavioral measures (e.g., iterated Prisoner’s Dilemma Game) of *Cooperativeness*. Second, although there were no significant gender differences observed in the current study, gender differences should be further examined in future studies. One previous VBM study found positive correlations between *Cooperativeness* and gray matter volumes in the frontal regions for females, but not for males [48]. The frontal regions also showed significant gender differences in gray matter volumes [48]. Thus, there are likely gender differences in the anatomical neural correlates of *Cooperativeness*. Third, the current study focused only on fiber connectivity without directly examining the functions of the relevant brain regions or functional connectivity among them. Future research in this area should integrate more brain measures. Finally, the voxels in the current DTI dataset were anisotropic, which did not allow for easy detection of specific smaller and bendier tracts [60, 61], that might be important for personality traits.

In summary, with probabilistic tracking of diffusion tensor images, the current study investigated the associations between *Cooperativeness* and fiber connectivity between the striatum and subcortical and cortical regions. After controlling for confounding factors such as gender and the proportional volume of gray matter relative to the total intracranial volume, results showed negative correlations between *Cooperativeness* and fiber connectivity from the caudate to the rostral CC and vlPFC. These findings provided support for the involvement of striatum-projected networks in *Cooperativeness*.

## Supporting Information

S1 Dataset Masks of ten brain regions from AAL map. (RAR)

S2 Dataset Group-average tractographic images from the striatum to nine target regions. (RAR)

S3 Dataset The resulted statistic maps for nine target regions. (RAR)

S1 Fig Locations of target regions in the current study. Abbreviations: Amy, amygdala; Hipp, hippocampus; mOFC, the medial orbitofrontal cortex; rostral CC, the rostral cingulate cortex; dorsal CC, the dorsal cingulate cortex; PCC, the posterior cingulate cortex/retrosplenial cortex; lOFC, the lateral orbitofrontal cortex; vlPFC, the ventrolateral prefrontal cortex; dlPFC, the dorsolateral prefrontal cortex.
(TIF)

S1 Table Scores of *Coorperativeness* and volumes of ten brain regions and extracted fiber connectivity. These data were used for Table 1. Abbreviations: Amyg, amygdala; Hipp, hippocampus; mOFC, the medial orbitofrontal cortex; rostral CC, the rostral cingulate cortex; dorsal CC, the dorsal cingulate cortex; PCC, the posterior cingulate cortex/retrosplenial cortex; lOFC, the lateral orbitofrontal cortex; vlPFC, the ventrolateral prefrontal cortex; dlPFC, the dorsolateral prefrontal cortex.
(XLSX)

## Abbreviations

TCI: the temperament and character inventory
DTI: diffusion tensor imaging
mOFC: the medial orbitofrontal cortex
lOFC: the lateral orbitofrontal cortex
vlPFC: the ventrolateral prefrontal cortex
dlPFC: the dorsolateral prefrontal cortex
PCC: the posterior cingulate cortex/retrosplenial cortex
dorsal CC: the dorsal cingulate cortex
rostral CC: the rostral cingulate cortex
Amy: amygdala
Hipp: hippocampus
ICV: intracranial volume
WM: white matter
GM: gray matter
