# Supplementary figures and images for: Striatum-Centered Fiber Connectivity Is Associated with the Personality Trait of Cooperativeness
Source: PLoS One. 2016 Oct 18;11(10):e0162160. doi: 10.1371/journal.pone.0162160 (PMC5068751; doi:10.1371/journal.pone.0162160)

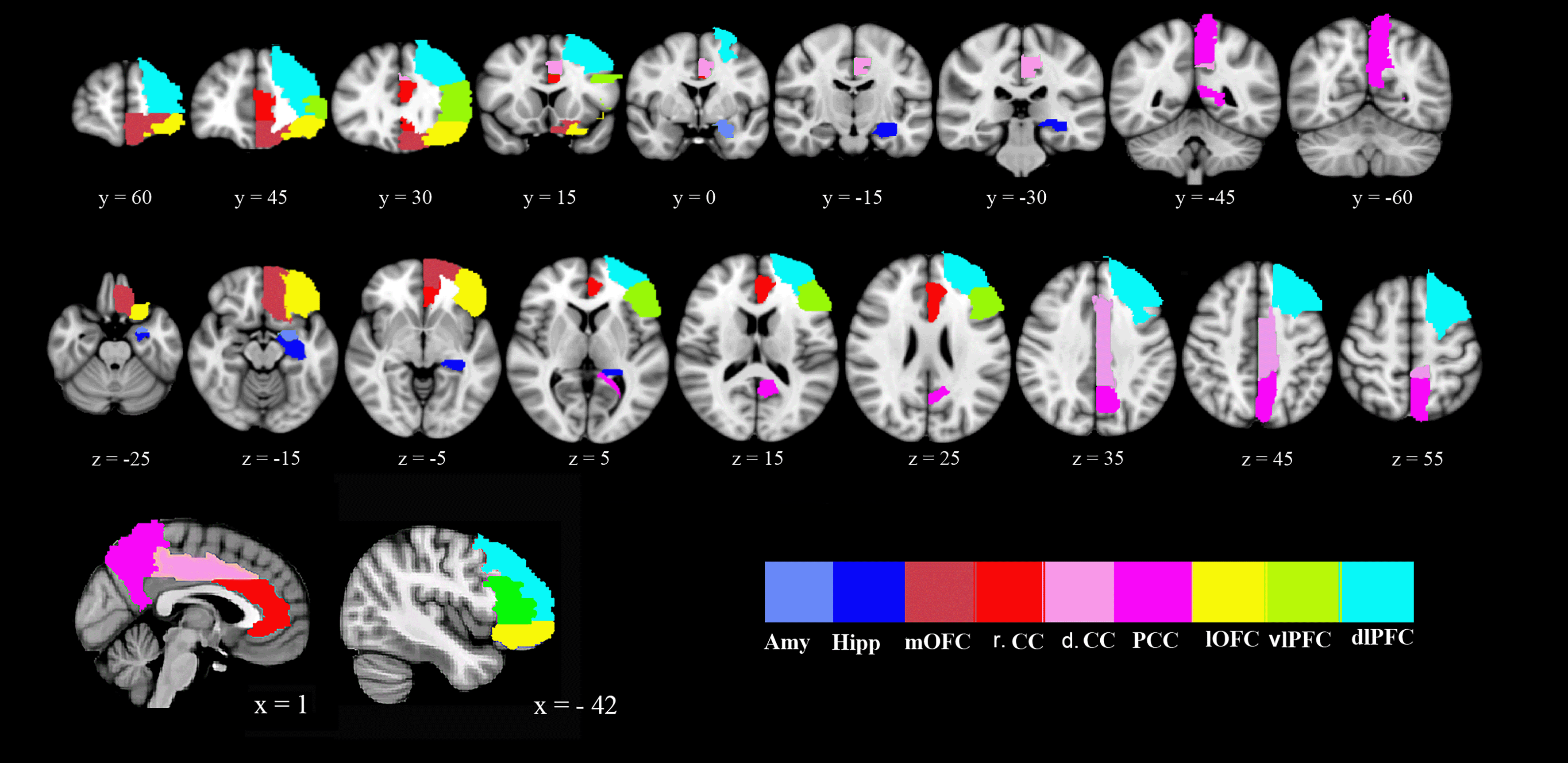

Supplement: S1 Fig — Abbreviations: Amy, amygdala; Hipp, hippocampus; mOFC, the medial orbitofrontal cortex; rostral CC, the rostral cingulate cortex; dorsal CC, the dorsal cingulate cortex; PCC, the posterior cingulate cortex/retrosplenial cortex; lOFC, the lateral orbitofrontal cortex; vlPFC, the ventrolateral prefrontal cortex; dlPFC, the dorsolateral prefrontal cortex. (TIF) [file pone.0162160.s004.tif]
